# On Fungous Tumours of the Meninges of the Brain

**Published:** 1830-10-01

**Authors:** 


XXVII.

### M. Cruveilhier on Fungous Tumors of the Meninges of the Brain.*

The study of cancerous diseases, though repulsive in itself, and not leading directly to the splendid goal of professional ambition, the cure of human maladies, is productive of many beneficial results. It teaches us that difficult lesson to learn, the when and the where to restrain our hands; it tells us the occasions on which interference would be mischievous, and heroic remedies pernicious; it informs the surgeon, that if there are cases adapted for the knife or the cautery, there are others which to meddle with is murder. We have had but too many opportunities of witnessing the disastrous consequences of operations performed upon malignant tumours, in an improper manner or at an improper time. About two years ago, a woman presented herself to a surgeon with a tumour upon the head. It was soft, had an opening in it formed by ulceration, and had existed for a few weeks only. The gentleman, thinking it a common encysted tumour, introduced a lancet and probe, which appeared to pass deeply, and poked about for some time to his own satisfaction, though not to that of the lookers on. A severe and strange set of symptoms followed, and in two or three days the patient was no more. On dissection, the tumour was found to be fungus hæmatodes, and to be in contact with the dura mater, having extended inwards through a large opening in the cranium. A few days ago, we saw a female with ulcerated scirrhus of the breast, who, according to her own account, had undergone a most improper operation in the country. Part of the breast had been removed, a part of the scirrhous tubercle being left behind, to grow and to ulcerate afresh. We might swell relations of this kind to a fearful magnitude, but enough has been said to establish the position with which we set out.

Malignant tumours on the head are not uncommon. In the paper by Sir Astley Cooper on exostoses, published in his and Mr. Travers' surgical essays, one or two cases of fungus hæmatodes are detailed. We have ourselves seen several, and M. Cruveilhier, in the 8th livraison of his Plates of Morbid Anatomy, before us, delineates some well-marked specimens of the disease. We believe that the origin of some of these tumours is incorrectly described by some authors. M. Louis, the celebrated secretary of the still more celebrated French Academy, conceived that these malignant tumours proceeded from the dura mater, and described them under the title of fungus of that membrane. Siébold attempted to overthrow the opinions of M. Louis, and, relying on a case or two, maintained that the fungus originated in the diploë of the cranial bones. From that time to this there have been many and hot controversies on the subject; but, like the men with the cameleon, all are right and all are wrong. It will be our object to prove that, oc-

---

* Anatomie Pathologique. Huitieme Livraison.

casionally, the malignant growth takes its rise from the dura mater, and occasionally also from the diploë. If we ventured to place reliance on our own observations, we should say that the latter description of cases was more frequent than the former: but we speak on this point with diffidence. The history of this controversy, is the type and the emblem of almost every dispute in physic. One man saw a case in which the fungus originated in the dura mater: another found an instance of its birth in the diploë. Each maintained that his was the real Simon Pure, each concluded that his adversary was mistaken. Like the knights in the fable, they lustily fought on their own sides of the shield, but, unlike those knights, they had not the good hap to be thrown to the opposite, and discover their respective mistakes. So it is with physic and physicians. A man has a case which bears on a mooted point: — through the medium of excusable delusion, and the glass of personal feeling, this solitary fact is erected into a principle—and for that principle he fights with as much pertinacity as if he were contending for the empire of the world. Poor worm! The observer of to-morrow disbelieves his fact and upsets his reasonings, to substitute a tissue as ephemeral in their stead.

M. Cruveilhier takes a sensible and extensive view of these cancerous tumours. He shews that they sometimes arise in the dura mater, sometimes in the diploë. Of the former there are two classes: the one take their growth from the external layer of the membrane, and protrude through the cranial bones; the second proceed from its internal surface, and press inwards on the brain. Other tumours, again, are formed in the cellular texture beneath the arachnoid, and, amalgamating that membrane to themselves, it becomes difficult to determine that they have or have not been produced from the internal surface of the dura mater. Lastly, it is not uncommon for tumours of the cranial bones to exist along with tumours springing from the interior of the dura mater. In the present article, M. Cruveilhier confines himself to the consideration of productions of the latter class.

We have already stated, that cancerous tumours attached to the interior of the dura mater are more frequent than those external to it. We meet them on the surface of the brain, at the basis, attached to the falx and the tentorium. When situated on the petrous portion of the temporal bone, they may rather be regarded as belonging to it than to the dura mater; when in the sella turcica, they have erroneously been considered as a disease of the pituitary gland or infundibulum.

These tumours frequently appear to follow a blow, a fall, or a concussion; the symptoms attending them are mostly equivocal; indeed in the earlier stages there are usually none at all. But, sooner or later, the pressure, gradually augmented on the brain, becomes a source of irritation to itself or its membranes, and a train of symptoms, sometimes sudden, sometimes slow, are the necessary consequence. Frequently hemiplegia suddenly comes on; we have seen a complete and rapidly fatal apoplexy. A man, says our author, sixty years of age, was returning on foot to his native place, when suddenly he lost his senses, and recovered with hemiplegia of the right side. Nearly four months afterwards he died in the Ste. Antoine Hospital; and, on dissection, a cancerous tumour, as large as the fist, was found at the side of the falx cerebri, beneath the cerebral arachnoid, and lodged in a deep excavation of the middle lobe of the brain. In the case of sudden apoplexy to which we have alluded, the patient, a middle-aged woman, had been subject, for some time, to obscure cerebral symptoms, prior to invasion of the fatal attack. On dissection, a tumour of fungus hæmatodes was found in the substance of the right hemisphere of the brain.

Most commonly, and especially when the tumour occupies some portion of the basis, the symptoms of compression are established more gradually; sensation and motion are first diminished and then destroyed in the parts of the body in connexion with the affected side of the brain; the intellectual faculties are enfeebled: and idiocy and hemiplegia are established. M. Cruveilhier thinks that this train of symptoms is sufficient to point out the nature of the case, but we fear he is too sanguine. Even in those who are attacked with sudden symptoms, he believes that severe pains in the paralysed limbs, epileptiform seizures, long-established pains in the head and disturbance of intellect before the occurrence of hemiplegia, somnolency, &c. may furnish an intelligible guide to correct diagnosis. We need scarcely observe to practical men that these criteria will often prove deceptive; but still we believe that, if a careful practitioner has his wits about him, he may not unfrequently prognosticate, from such data, the nature of the disease. It is well ascertained that these cancerous tumours are seldom in themselves the cause of death. The fatal termination is induced by serous effusion between the membranes, arachnitis, more frequently still an apoplectic extravasation of blood, or extensive contiguous ramollissement. An important point is the difference of effect produced by disease of the same intensity in different individuals. As this, however, is little susceptible of satisfactory investigation, we leave it to those who have leisure or talent for unravelling the mysterious doctrine of susceptibility of habit. No doubt the position of the tumour and the direction which it takes must exercise influence in determining the relative quantum of mischief. With these remarks we proceed to the detail of particular cases. These are illustrated by coloured lithographic plates of the morbid lesions, conveying their nature to the eye in a very clear manner; if not so highly finished as some in this country, they are equally faithful, and, by reason of their cheapness, more generally useful.

CASE 1. *Hemiplegia—Epileptic Convulsions—Cancerous Tumor of the Dura Mater.*

Lecouvreur, æt. 65, was brought to the Maison de Santé in August, 1829, with hemiplegia of the right side. There were also severe pains throughout the body, and attacks like epilepsy occurred at long intervals. The intellectual faculties continued unimpaired, and the patient died at length worn out with sloughs on the sacrum and trochanters, the consequence of pressure.

*Sectio Cadaveris:*—On raising the scalp a considerable tumour was discovered over the parietal, and near the coronal suture. Its texture was spongy with bony spiculæ intermixed; it extended through the cranium to the dura mater over the superior longitudinal sinus. The inner table of the skull was in part absorbed, the tumour extended still farther in the diploë, and on slitting open the longitudinal sinus it was found to contain some small végétations of medullary matter. Besides this, two roundish cancerous bodies, grew from the dura mater in the angle between it and the falx; they pressed upon the brain, and the larger had occasioned a deep excavation in the left hemisphere, whilst the smaller was lodged in a similar manner in the right. The convolutions beneath had disappeared, and in the left hemisphere the contiguous white cerebral matter had undergone a remarkable gelatinous ramollissement.

A very good plate of the disease is appended, together with some learned observations on the case. It is enough to remark that it offers an example, both of medullary tumour growing from the inner surface of the dura mater, and arising in the cranial diploë.

CASE 2. *Medullary Tumour growing from the falx, in a patient recently lithotomized.*

M. R. ætatis 65, having undergone lithotrity without success, was, sometime afterwards, attacked with a cerebral affection, succeeded by paraplegia of the lower extremities. In spite of M. Dupuytren's objections, he insisted on submitting to lithotomy, which was done on the 13th of August last. Cerebral symptoms supervened, and the patient died on the 16th.

*Sectio Cadaveris.* A large quantity of serum flowed from between the membranes; the brain itself was healthy. On the anterior extremity and right side of the falx, was "a fibrous tumour," the size of a large nut, lodged in a corresponding depression of the hemisphere. The arachnoid was healthy. The kidneys were large and soft. There were three sacculated stones in the bladder, and several little hollows capable of containing calculi. The cerebral tumour itself was hard, almost cartilaginous, and yielded on pressure a milky juice.

CASE 3. *Fungus of the internal surface of the Dura Mater—torpor—diminution of voluntary motion.*

A woman, ætatis 45, complained habitually of head-ache, and shortly before her admission into the Maison de Santé, was totally unable to walk. On admission, in September, 1829, her symptoms were, constant reclination on her back—immobility both when asleep or awake—involuntary discharge of urine—constipation. The left inferior extremity appeared to be weaker than the right; the left commissure of the lips to be depressed; she spoke but gruffly and with slowness; and she complained of pain in the forehead. The pulse was feeble, but varied in frequency. M. Cruveilhier suspected some tumour within the head. She died suddenly on the 3d of October.

*Sectio Cadaveris.* A tumour reached from the inferior surface of the dura mater to the right of the falx cerebri, lodged in a depression in the anterior part of the right hemisphere of the brain. The depressed convolutions were atrophied, but not disorganized. The tumour was knobbed, traversed by numerous vessels, soft in texture, and consisted of a pulpy substance with innumerable granulations disseminated through it.

CASE IV. *Fungus from the interior of the Dura Mater, penetrating into the nose.*

A man, about 30 years of age, was affected with perfect amaurosis, from which he recovered for one day, but immediately relapsed. He died suddenly and unexpectedly. On dissection, the inferior and internal parts of the anterior lobes of the brain were converted into a tubercular mass, which had destroyed the cribriform lamella, and entered the nasal fossæ and the æthmoid cells. No vestige remained of the olfactory nerves, the optic commissure was pressed upon, and the orbital portion of the optic nerves was reduced to little else than neurilema. It was *said* that the patient complained of the fetor of pus, and took tobacco with pleasure.

CASE V. *Fungus on the surface of the Brain—sudden Hemiplegia—Epileptic seizure.*

J. D. Glaber, ætatis 66, had long complained of head-aches and debility, when in June, 1829, he was suddenly attacked with hemiplegia on the left side. On the 15th of August, he was seized with violent convulsive motions, confined to the paralysed limbs, which lasted for half an hour. Debility made progress, slight stupor succeeded, and on the 9th of September he expired, retaining all his consciousness to the last.

*Sectio Cadaveris.* Beneath the arachnoid, on the surface of the right hemisphere, near the union of the anterior and middle lobes, was a fungous tumour the size of a nut. The brain was pressed on, and its convolutions destroyed; around it presented the yellow ramollissement.

This completes the list of cases related by M. Cruveilhier. We have abridged them considerably, but probably our readers will not quarrel with us for that. We have given sufficient details to shew that the symptoms will vary in different individuals, but still the majority suffer from head-ache, partial palsy, or other slight cerebral symptom, before the hemiplegia, or the coma, or the sudden apoplectic seizure supervene. If these circumstances be considered with care, if the patient have a more than ordinarily sallow aspect, and if his age be rather advanced, the practitioner may frequently be enabled to prognosticate the existence of a tumour within the cranium. We have said that the age is commonly advanced, and for proof of the assertion we appeal to facts. In the present series of five cases, three patients were above sixty, and the youngest was thirty. We should mention before we quit the subject, that all the cases are accompanied in M. Cruveilhier's valuable work with coloured plates depicting the particular lesion.

---

